# Extraction with SPME and Synthesis of 2-Methyl-6-vinylpyrazine by a ‘One Pot’ Reaction Using Microwaves 

**DOI:** 10.3390/molecules14062160

**Published:** 2009-06-15

**Authors:** Norma Robledo, Jaime Escalante, René Arzuffi

**Affiliations:** 1 Centro de Desarrollo de Productos Bióticos, IPN. Km. 8.5 Carretera Yautepec-Jojutla Yautepec, Morelos, C.P. 62731, Mexico; 2 Centro de Investigaciones Químicas, Universidad Autónoma del Estado de Morelos. Av. Universidad No. 1001, Col. Chamilpa, C.P. 62210 Cuernavaca, Mor., Mexico

**Keywords:** SPME, Hoffman methylation, microwave, 2-methyl-6-vinylpyrazine, sexual pheromone

## Abstract

A synthesis of 2-methyl-6-vinylpyrazine was carried out by way of a ‘one pot’ reaction. In order to establish the efficiency of this synthesis the extraction of the volatiles released by male papaya fruit flies was performed by SPME (solid phase micro-extraction). The compound was separated and identified using GC/MSD (gas chromatography/mass spectrometry detector).

## Introduction

**Figure 1:**
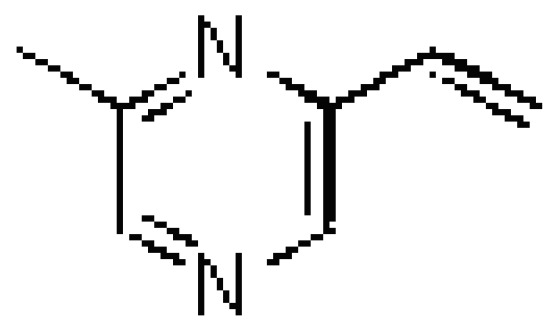
2-Methyl-6-vinylpyrazine.

Sexual pheromones are chemical signals that regulate the sexual behavior of insect species and which typically consist of characteristic components in specific proportions. These compounds are released into the air during sexual calling and can function at a distance, inducing orientation or provoking attraction responses and courtship in the opposite sex [1]. 2-Methyl-6-vinylpyrazine (Figure 1) is the main component of the *Toxotrypana curvicauda* (Gerstaecker) sexual pheromone [2]. This fly is a major pest of the papaya fruit, and 2-methyl-6-vinylpyrazine has been used for management of this fly in Florida USA and Central America [1]. 2-methyl-6-vinylpyrazine has been reported in various products, as part of the Chinese liquor aroma pyrazine [3], in coffee [4], soy [5], maize flour [6] and wheat flour [7].

Previously this component was obtained using a ‘traditional’ extraction method, volatiles were extracted from the charcoal filter trap with dichloromethane and evaporated under vacuum [2]. The pyrazine was identified and synthesized using the method of Kamal using a Hoffman exhaustive methylation route in three sequential steps [2]: a) the production of 2-methyl-6-dimethylaminoethyl-pyrazine from 2,6-dimethylpyrazine, dimethylamine hydrochloride, and formaldehyde (under reflux); b) the conversion of the quaternary salt with methyl iodide (6-methylpyrazylethyltrimethylammonium iodide) and finally c) preparation of 2-methyl-6-vinylpyrazine with a total yield of 24%. Reflux heating took place over a total of 3.5 h; a stabilizing process of approximately 12 h was required for the production of the second intermediate. The purification and analysis of the intermediary products was necessary in this synthesis. Another reported synthesis of vinylpyrazines involves the chlorination of dimethylpyrazine to obtain the corresponding monocloromethyl isomer, and Wittig reaction after obtaining a phosphonium salt [8].

Currently the application of solid phase microextraction (SPME) for volatile compounds analysis has been studied [9]. SPME is a simple, selective technique, which takes only a short time to complete and uses no solvent. It can be applied to flavor volatiles analysis [9] and volatiles released for live insects. ****

The aim of this study was extract and identify 2-methyl-6-vinylpyrazine from calling male emissions and carry out a synthesis of this pyrazine by means of a ‘one pot’ reaction so as to achieve an improved yield, reduce the reaction time and avoid the purification and evaluation of intermediates which is required in the previously described procedure.

## Results and Discussion

### Identification of 2-methyl-6-vinylpyrazine in male emissions

**Figure 2:**
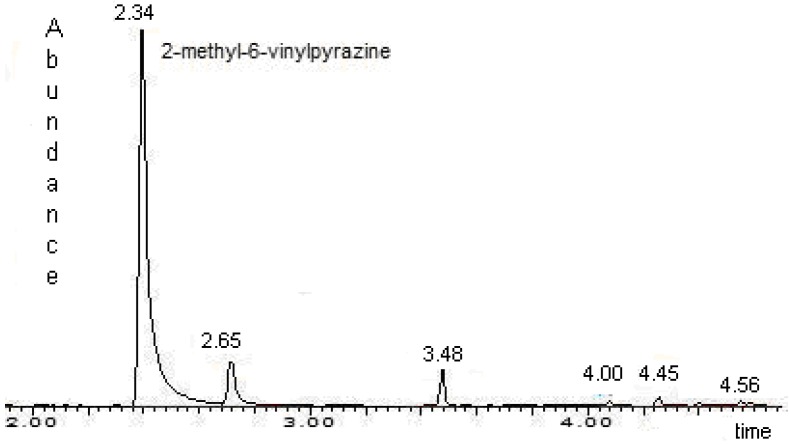
Gas Chromatogram of volatiles released for male using SPME.

In this study 2-methyl-6-vinylpyrazine was extracted and identified in the volatiles released from 3 and 7 day old adult males using SPME and gas chromatography/mass spectrometry detector (GC/MSD). Previously reported 2-methyl-6-vinylpyrazine with a retention time (RT) 2.34 ± 0.05 min was identified as the principal component of the mixture [2] (Figure 2). It was possible to capture pyrazine in larger quantities than those previously reported by Chuman *et al*. [2] who performed emission extraction from individually placed 3-5 day old males using a charcoal filter trap, and volatiles were extracted with dichloromethane, obtaining an average of 63.2±33.2 ng/male-h. However, as regards 3 and 7 day old males, 670 ±120 ng/male-h and 950 ±130 ng/male-h were captured respectively. Thus SPME gave direct individual results, capturing a greater quantity of pyrazine and reducing standard error of measurements, obtained 18% and 14% of the average for 3 and 7 days respectively, compared to 53% of the average with charcoal filter trap [2]. The chemical composition of the polydimethylsiloxane/divinylbenzene (PDMS/DVB) fiber absorbs mainly nitroaromatic volatile compounds (PM of 50-300) resulting in a higher affinity to 2-methyl-6-vinylpyrazine from male emissions and thus a more specific and selective capture than in the procedure reported by Chuman *et al*. [2].

### Synthesis of 2-methyl-6-vinylpyrazine

**Scheme 1:**
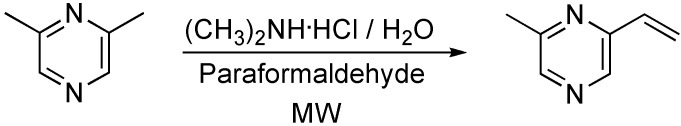
Synthesis of 2-methyl-6-vinylpyrazine.

The synthesis of 2-methyl-6-vinylpyrazine was performed by means of a one step Hoffman methylation under microwave heating (Scheme 1). As has been reported for other compounds, the use of this methodology allows the speeding up of chemical reactions [10], resulting in substantial reductions in reaction times [10], the production of some products with microwaves that could not be obtained with conventional heating [11], a variety of chemical transformations [12], increases in the yield and a reduction in solvents and waste [12,13], when compared with traditional methods [2,13,14]. Lacking sequential reactions (step by step) there is no need to purify intermediates [15], as in the case of triazolo pyrazines [16] and preparation of *N*-(alkoxycarbonylmethyl) nucleobases [17]. 

Finally the chromatographic/spectrometric analysis indicated that the synthesized substance was 2-methyl-6-vinylpyrazine produced in a RT of 2.36 ± 0.05, Retention Index (RI) 1015 (RI Literature 1016) [3], C_7_H_8_N_2_, Mol. Wt. 120.15, CAS 13925-09-2, with 90% purity. Analysis by ^1^H- and ^13^C- NMR spectroscopy indicated that the product corresponded to 2-methyl-6-vinylpyrazine. This compound was used as a reference standard. The use of microwaves as an energy source was very efficient, demonstrating a noteworthy advantage over traditional thermal methods [13,14]. Yield was higher than previously reported [2] and pyrazine purity was equivalent to that obtained by Chuman *et al.* [2].

## Conclusions

2-Methyl-6-vinylpyrazine was extracted directly by SPME from calling male emissions, greatly increasing extraction efficiency when compared with the previously used method. Moreover, a simple, efficient and fast ‘one-pot’ synthesis of this pyrazine was carried out. 

## Experimental

### General

All the reagents and solvents employed were purchased from Aldrich and used without further purification. For microwave irradiation we used a monomode microwave CEM Discover apparatus. ^1^H-NMR and ^13^C-NMR spectra were recorded in deuterated Chloroform (CDCl_3_) solutions with TMS as internal standard on Varian Gemini 200 MHz spectrometer. Mass spectra were recorded on a GC HP6890-MSD HP5972.

### Insects

*T*. *curvicauda* larvae were obtained from parasitized papaya fruit from a small plantation located in Yautepec, Morelos, México (coordinates 18°05’ north, 99°03’ west; altitude of 1,100 m). The larvae were placed in 500 mL plastic containers with soil from the original collection site and once the adults emerged were maintained in a breeding chamber (25°C temperature, 50-60% relative humidity and a 12-12 h light-dark cycle). Recently emerged males were used in all experiments and were kept separate with sufficient water and sugar for food until experiment completion. 

### Extraction and identification of volatile compounds

*Volatile collection using solid phase microextraction*: 3 and 7 day old males (N= 9, by age) were individually placed in 40 mL transparent glass vials with screw tops and PTFE/silicon septum (27089-U, Supelco, USA). The volatile compounds released during sexual calling were collected using SPME with a 65 μm fiber PDMS/DVB (57326-U, Supelco, USA) during three hours at 25°C, at 12-15 h. Once the compounds were captured an analysis was performed using GC/MSD equipped with a HP 5 MS non-polar column (30 m, 250 µm internal diameter and 0.25 µm film thickness) (Agilent, Palo Alto, CA, USA). Before analysis, the fiber was conditioned by inserting it into the GC injector at the 250°C for 30 min to prevent contamination.

*Chemical analysis and identification of volatile compounds*: The chemical analysis of volatile compounds absorbed by the fiber (SPME) and synthetic 2-methyl-6-vinylpyrazine (injection volume 1 µL), took place in a GC/MSD. The oven starting temperature was 60°C, increasing by 30°C/min until reaching 200 °C, a temperature maintained for 2 min. Helium was used as a carrier gas at a constant flow of 1 mL/min. The injection temperature was 250 °C and the auxiliary temperature was 280 °C. The injector worked in splitless mode during 0.30 min. The MSD used electronic ionization (70 EV), in SCAN mode and with a 35 to 550 UMA, provided by a spectral library (Wiley175, NIST). Compounds were identified considering retention times, retention index, mass spectral evaluation and comparing with the spectral library. 

**Figure 3:**
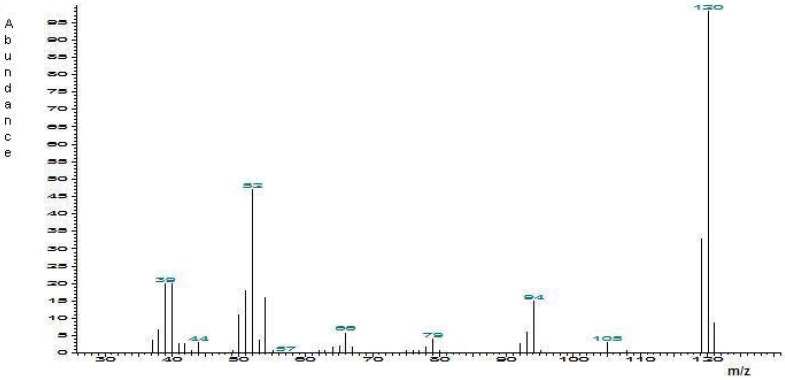
Mass spectrum of 2-methyl-6-vinylpyrazine.

*2-Methyl-6-vinylpyrazine synthesis*: A suspension of 2,6-dimethylpyrazine (0.32 g, 3 mmol), dimethylamine hydrochloride (0.24 g, 3 mmol), paraformaldehyde (0.105 g, 1.16 mmol), and water (2 mL) was placed in a 10 mL glass microwave reaction vessel containing a stir bar. The reaction vessel was sealed with a cap and then placed into the microwave cavity. The microwave unit was programmed to heat the reaction mixture at 100 °C with 100 Watts power for 2 h. After the reaction was completed and the vessel was cooled to below 50 °C using a flow of compressed air, water (5 mL) was added and the crude material was extracted with ether (10 mL x 5), and the organic phase was dried with magnesium sulfate. The resulting material was filtered and concentrated in a rotavapor, producing 280 mg of reaction crude, which was then purified by column chromatography using ether as solvent, to afford 0.14 g (38% yield) as a colorless oil. ^1^H-NMR (200 MHz, CDCl_3_) δ 2.56 (s, 3H), 5.60 (dd, *J* = 1.2 and 11.0 Hz, 1H), 6.33 (dd, *J* = 1.2 and 17.4 Hz, 1H), 6.79 (dd, *J* = 17.4 and 11 Hz, 1H), 8.30 (s, 1H), 8.40 (s, 1H); ^13^C-NMR (50 MHz, CDCl_3_) δ 21.7, 120.5, 133.7, 139.7, 141.3, 150.0, 153.3. The synthesized standard (1 μg/mL) was analyzed with chromatographic-spectrometric analysis. The mass spectral data were (m/z): 39 (21%), 52 (48%), 54 (18%) 94 (13%), 119 (33%), 120 (100%) (Figure 3).

### Quantitative Analysis

In order to compare the extraction method used in this experiment with the one previously reported [2] a calibration curve was drawn using the reference standard at the following concentrations: 187, 375, 1126, 1500 and 3500 ng.

Applying the equation:

Y = a+bx; parameters, a = 7.45667e+006, b = 2.0026e+007.
